# Impact of the Interfacial Kapitza Resistance on Colloidal Thermophoresis

**DOI:** 10.1021/acsomega.4c06427

**Published:** 2024-10-17

**Authors:** Juan D. Olarte-Plata, Fernando Bresme

**Affiliations:** Department of Chemistry, Molecular Sciences Research Hub Imperial College, Imperial College London, London W12 0BZ, U.K.

## Abstract

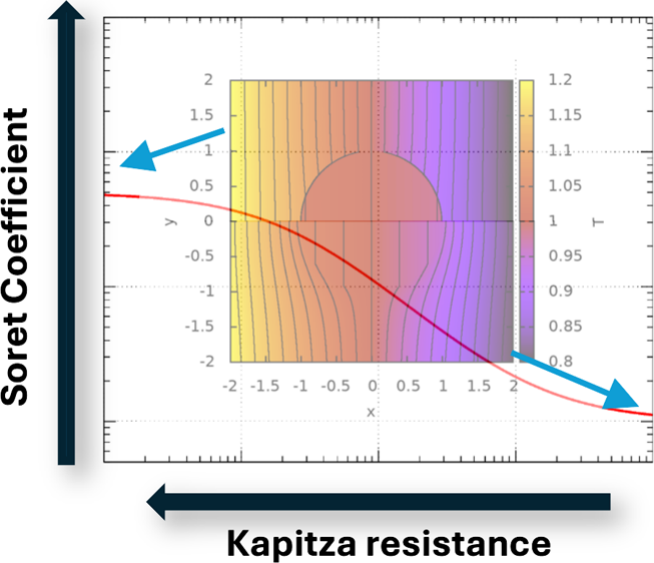

Thermal gradients impart thermophoretic forces on colloidal particles, pushing colloids toward cold or hot regions, a phenomenon called thermophoresis. Current theoretical approaches relate the Soret coefficient to local changes in the interfacial tension around the colloid, which lead to fluid flow around the colloid surface. The Kapitza resistance, a key variable in the description of interfacial heat transport, is an experimentally accessible property that modifies interfacial thermal fields. Here, we introduce a theoretical approach that describes colloid thermophoretic forces by incorporating explicitly Kapitza resistance effects. Our formulation can be used to monitor the dependence of thermophoresis on interfacial thermal resistance. We show that the resistance modifies the thermal field around the colloids and identify experimental conditions where the Kapitza resistance influences the thermophoretic forces. We validate our theoretical approach by implementing a nonequilibrium molecular dynamics model of a colloid suspended in a solvent.

## Introduction

Thermophoresis describes the motion of colloidal particles in solution. This physical effect was discovered by Ludwig and Soret in the 19th century using alkali halide aqueous solutions.^1,2^ Thermophoresis is a complex nonequilibrium phenomenon whose explanation has motivated experimental and theoretical works.^3−15^ Following the behavior observed in thermodiffusion, colloids can feature thermophobic/philic behavior at high/low temperatures. The phobic/philic transition temperature depends on the particle size^16^ and screening length, in charged colloids.^8^ Duhr and Braun introduced the idea of solvation entropy, connecting thermophoresis to interfacial properties.^8^ Würger^6^ developed a hydrodynamic theory where the colloid thermophoresis depends on the solvent and colloid thermal conductivities, the temperature derivative of the solvent-colloid surface energy (surface entropy) and the solvent viscosity. Arango-Restrepo and Rubi^13^ derived an equation for the Soret coefficient using the Faxén theorem. Their equation does incorporate the derivative of the interfacial tension with temperature and the viscosities of the fluid and the particle.

Additional work on fluid–solid interfaces has been performed in the context of confined fluids and surfaces^17−20^ focusing on the thermo-osmotic coefficient and following nonequilibrium thermodynamics theory.^21−24^ The fluid flow induced by thermo-osmosis emerges from stresses along a substrate fluid interface, and it is therefore an interfacial phenomenon. Evidence for fluid flow around nanoparticles has been provided recently using computer simulations.^15,25^

At the colloid-fluid interface, thermodynamic and transport properties, such as density and thermal conductivity, feature abrupt changes. In the presence of a thermal gradient, these discontinuities give rise to the Kapitza resistance^26−28^ and an interfacial temperature “jump” that might influence the thermal field around a colloid, and potentially thermophoresis. The importance of the Kapitza resistance, or its inverse, the Interfacial Thermal Conductance (ITC), *G*_K_, will depend on the magnitude of the conductances and, therefore, the colloid-solvent interfacial properties. High ITC will result in small temperature “jumps” at the colloid surface, but low ITC might lead to important differences in the interfacial temperatures of the colloid and the solvent. To understand what constitutes high or low ITC, it is instructive to examine experimental studies of hydrophobic and hydrophilic interfaces.^29^ The reported ITCs vary between *G*_K_ = 50 MW/(K m^2^), “low”, and 150 MW/(K m^2^), “high” ITC. Computer simulations of liquid–vapor interfaces reported even lower ITCs, ∼1 MW/(K m^2^),^30^ or higher ∼200–300 MW/(K m^2^)^31,32^ for gold-water interfaces. At low ITC, *G*_K_ ∼ 10 MW/(K m^2^) measurable temperature differences between solvent and colloid, Δ*T* ∼ 0.1–1 K, might appear for heat fluxes achievable in microfluidics (∼10^6^ K/m)^8^ or plasmonic heating (10^8^ K/m).^33^

Here we incorporate ITC effects in the theory of thermophoresis. The ITC is an experimental variable that is becoming increasingly important in the design of thermal management devices, as well as on the interpretation of nanoscale heat transfer experiments.^34,35^ We show that the ITC modifies the temperature field around a colloid, and the thermophoretic force, particularly at low ITCs. We corroborate the general predictions of the theory using nonequilibrium molecular dynamics simulations.

## Results and Discussion

### Theoretical Model

Several authors^6,36−38^ derived equations for the Soret coefficient of spherical colloids by considering the deformation of the temperature field and the ensuing interfacial tension gradient around the colloid, which leads to a Marangoni force. The deformation of the temperature field emerges from the contrast of thermal conductivities of the solvent, κ^s^, and that of the colloid, κ^c^. For a spherical colloid immersed in an external temperature field ∇*T*, of magnitude |∇*T*| in the *x⃗* direction, the temperature profile (in polar coordinates), far away from the particle is
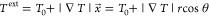
1where *T*_0_ is a reference temperature far from the colloid. Near the colloid, the solvent, *T*^s^(*R*, θ) and colloid, *T*^c^(*R*, θ) temperature profiles fulfill the boundary conditions:
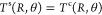
2
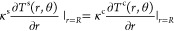
3

Following the solution of the Laplace equation^36−38^ the temperature profiles are given by,
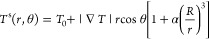
4
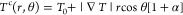
5where *R* is the colloid radius. The parameter α quantifies the thermal conductivity contrast between the colloid and the fluid. An explicit equation for α follows from the boundary condition in eq 3,
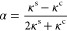
6

The solvent-colloid interface results in an interfacial thermal conductance that modifies the boundary conditions given by eqs 2 and 3, as the temperature features a discontinuous jump, Δ*T* = *J*_q_/*G*_K_, defined by the heat flux, *J*_q_, and the interfacial thermal conductance, *G*_K_. The new boundary conditions, including *G*_K_ are,
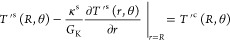
7
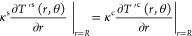
8where the prime indicates the equations include the interfacial thermal conductance effect. The solution of eqs 7 and 8 gives the corresponding temperature profiles,
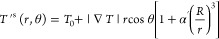
9
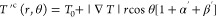
10where α′ and β′ are
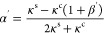
11
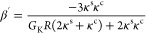
12

For *G*_K_ → *∞*, β′→ 0 and we recover eqs 4 and 5. We note that eqs 9 and 10 agree with those derived in ref (39) to obtain the effective thermal conductivity of composites.

**Figure 1:**
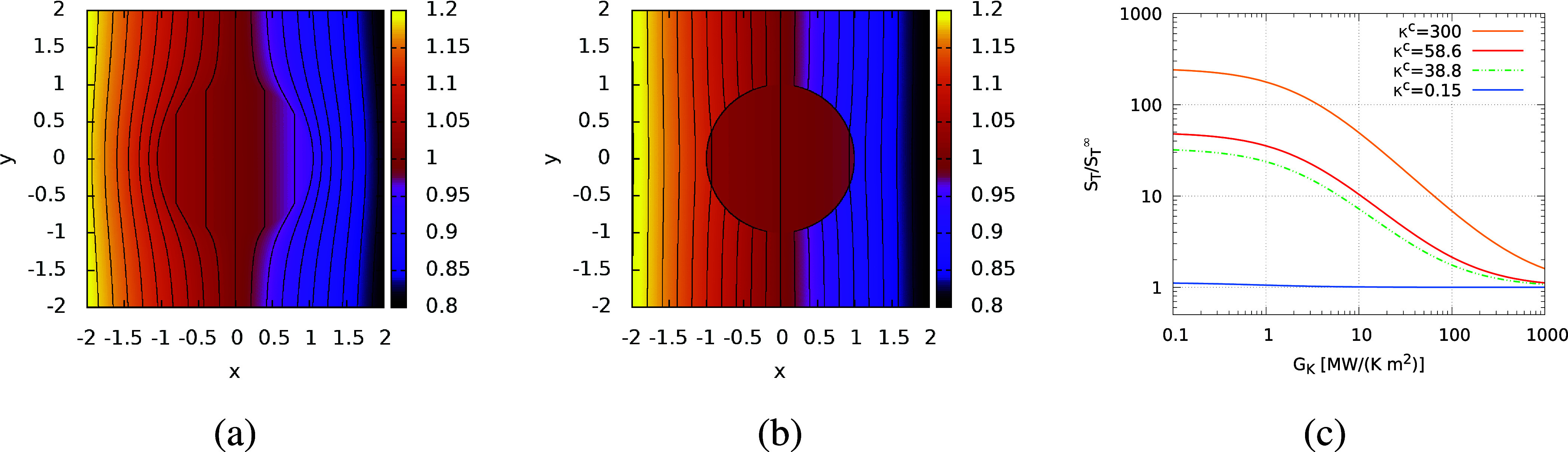
Temperature field around a spherical colloid of radius *R* = 1.0, without (a) and with (b) ITC effects. The results in (a) were obtained using eqs 4 and 5, and those in (b) with eqs 9 and 10. We used κ^s^ = 1.0, κ^c^ = 4.0, *G*_K_ = 1.0, average temperature *T*_0_ = 1, and |∇*T*| = 0.1. These reduced units (250 nm for length, 300 *k*_B_ for energy and 10^–16^ s for time) are compatible with a colloid of radius 250 nm in water at 300 K, κ^c^/κ^s^ = 4 (κ^s^ = 0.6 W/(K m)), *G*_k_ ∼ 3 MW/(K m^2^). (c) Predicted *S*_T_ as a function of *G*_K_, for different κ^c^ (in units of W/(K m)) and κ^s^ = 0.6 W/(K m). The lines represent results obtained with eq 17. *S*_T_ has been divided by the Soret coefficient, *S*_T_^∞^ at *G*_K_ → *∞*, i.e. when the ITC effects are neglected. The results for *S*_T_^∞^ were obtained using eq 15 and α (eq 6) instead of α′. Note that the isolines in panel (b) do not cross around *x* = −1, 1. The apparent crossing appears because the interfacial thermal resistance induces a discontinuity of the temperature profile at the particle surface.

Figure 1 shows the temperature profiles around the colloid with and without interfacial thermal conductance effects. The ITC has a significant impact on the temperature field, and the temperature profile features a discontinuity for the solution that includes the ITC effects. Although the temperature field appears less deformed in the vicinity of the colloid, the ITC leads to different thermal fields far from the colloid surface. The changes in the thermal field influence the thermophoretic force, thermophoretic velocity, and ultimately the Soret coefficient. We now follow the approach introduced in ref (6) to address these changes. We obtain the thermophoretic velocity by considering the surface stress around the colloid. The drift velocity of the particle is given by (see section 2 in the SI for a derivation of the equations given below):
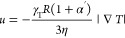
13where η is the solvent viscosity and γ_T_ = dγ/d*T* quantifies the change of the interfacial tension, γ, with temperature. In this theory, the thermophoretic force is a Marangoni force emerging from the surface stress around the colloid due to the temperature gradient. The thermal diffusion coefficient is defined by the factor in front of the thermal gradient in eq 13
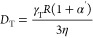
14

which again reduces to the equations reported in previous work^6^ when *G*_K_ is neglected. The Soret coefficient is given by *S*_T_ = *D*_T_/*D*, where *D* is the interdiffusion coefficient approximated for highly diluted suspensions by the colloid diffusion coefficient. For a spherical colloid, *D* = *k*_B_*T* /(ξπη*R*), where ξ is a numerical parameter accounting for the boundary conditions. The Soret coefficient is given by
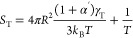
15where the α′ parameter considers the different thermal conductivities of the solvent and colloid and the interfacial thermal conductance. The second term of eq 15 represents the ideal contribution to the Soret coefficient. The origin of the factor “4” in eq 15 is justified in the derivation included in the SI (see section 3).

The scaling of the Soret coefficient with the square of the colloid radius (*R*^2^) and the inverse of the temperature is consistent with previous computations of Soret coefficients.^40^ However, α′ introduces an additional dependence on the colloid radius through eq 12. The solution, including *G*_K_ for the solvent and colloid temperature profiles, results in a larger deformation of the temperature field far from the particle (see Figure 1a,b) and a temperature discontinuity at the colloid surface. Based on eq 12, we expect that the impact of the interfacial thermal conductance on the Soret coefficient will be stronger when the thermal conductivities of the solvent and the colloid are very different. Figure 1c illustrates the impact of *G*_K_ on the predicted Soret coefficient for a colloid of radius *R* = 250 nm immersed in water with κ^s^ = 0.6 W/(K m). For the colloid, we consider a range of thermal conductivities, κ^c^ = 0.15 ··· 300 W/(K m). The intermediate thermal conductivities, 58.6 and 38.8 W/(K m), might be representative of the thermal conductivity of a gold nanoparticle coated with a 1 or 2 nm alkane passivating layer, with a thermal conductivity for the layer of 0.45 W/(K m)^41^ (see SI section 7, for more details). We also consider the thermal conductivity of a colloid made of polystyrene with κ^c^ = 0.15 W/(K m) as well as high thermal conductivity, of the order of that of bulk gold, κ^c^ = 300 W/(K m). The range of ITCs represented in Figure 1c, covers values corresponding to liquid–vapor interfaces 1 MW/(K m^2^),^42^ and hydrophobic and hydrophilic self-assembled monolayers (50–1000 MW/(K m^2^)).^29,41^ For similar solvent and colloid thermal conductivities, the impact of *G*_K_ is relatively small, and the results converge to the previous solution that assumes *G*_K_ → *∞*. The convergence is much slower when κ^s^ and κ^c^ are very different. Moreover, at low *G*_K_, the difference in the Soret coefficient is significant, even for interfacial thermal conductances corresponding to hydrophobic layers, ∼ 50 MW/(K m^2^). In summary, our results show that low ITCs can significantly enhance the Soret coefficient when the thermal conductivity of the colloid is much higher than the thermal conductivity of the solvent.

We have shown that the Soret coefficient is proportional to the parameter 1 + α′. We now extend the analysis of the dependence of this parameter on the thermal transport coefficients of the system by introducing the dimensionless quantities λ = κ^s^/κ^c^ and ϵ = *G*_K_*R*/κ^s^. The first quantity is simply the ratio between the thermal conductivities of the solvent and the colloid, while the latter relates the interfacial thermal resistance of the interface with that of the fluid. Using these definitions, we can express the parameter 1 + α′ as
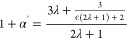
16

The solution when ϵ → *∞* converges to 1 + α = 3λ/(2λ + 1) = 3κ^s^/(2κ^s^ + κ^c^). The ratio between the solutions with and without considering the ITC can be written as
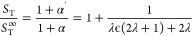
17where *S*_T_^∞^ is the Soret coefficient for *G*_K_ → *∞*.

### Test of the Theoretical Model Using Computer Simulations

**Figure 2:**
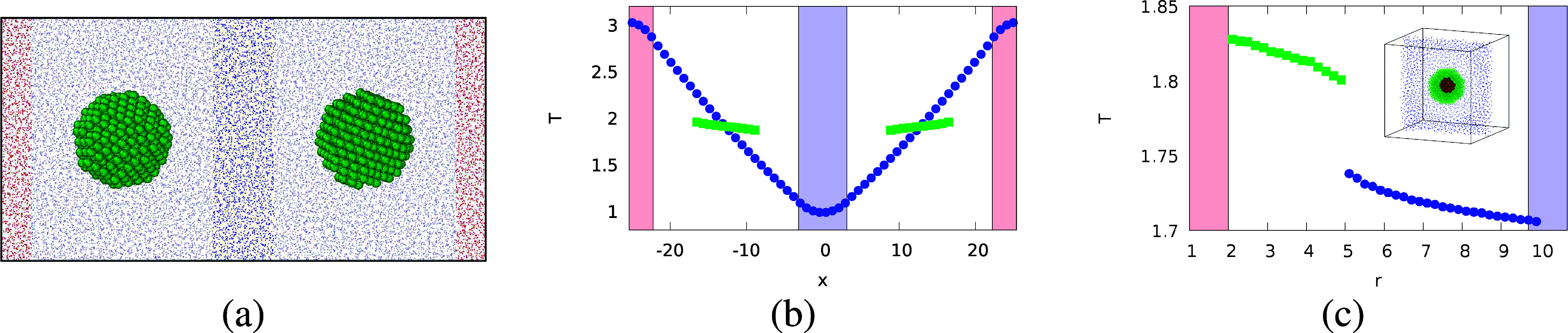
(a) Snapshot of the simulation box in the *external gradient* setup, showing the thermostatting regions (red-hot, blue-cold). The solvent particles are represented as blue dots for visualization purposes, and the colloid as green spheres. (b) Temperature profile for a system with ϵ_c_/ϵ_s_ = 20, showing the temperature of the fluid (blue circles) and the colloid (green squares). (c) Temperature profile for the *radial heat flux* system with ϵ_c_/ϵ_s_ = 20, showing the temperature of the fluid (blue circles) and the spherical particle (green squares), and the corresponding temperature jump. The inset shows a snapshot of the simulation box, highlighting the thermostated core (red) and the surrounding solvent (blue dots).

We test the theoretical predictions using computer simulations of a colloid immersed in a WCA solvent at reduced density, ρ = 0.8 (see SI for details on the simulation model and Figure 2 for the simulation set up). We also use the WCA potential to model the fluid-colloid interactions. The interactions inside the colloid are described with the spherically truncated and shifted Lennard-Jones potential with interaction strengths varying between ε_c_/ε_s_ = 20 ··· 100, where ε_s_ and ε_c_ are the interaction strengths between solvent particles and the particles in the colloid, respectively.

We used two simulation set-ups to test the theoretical predictions. *G*_K_, κ^s^ and κ^c^ were obtained using a *radial heat flux* simulation setup (see Figure 2c). The deformation of the temperature field due to the presence of the spherical particle was studied using an *external gradient* setup (see Figure 2a,b). In both set-ups, the thermal gradients are simulated explicitly by setting hot and cold boundaries (see Figure 2). The *external gradient* setup was also employed to compute the thermal conductivity of the solvent and the Soret coefficient by computing the thermophoretic force associated with the displacement of the colloids attached with a harmonic spring to the geometric center of the reservoirs shown in Figure 2a,b (see e.g. ref (15) for details on this method). The thermal conductivity and ITC were obtained from the heat flux using Fourier’s law, *J*_q_ = −κ ∇*T* and the Kapitza relation, *J*_q_ = *G*_K_Δ*T*, where ∇*T* is the thermal gradient, and Δ*T* the temperature “jump” at the colloid-solvent interface (see Figure 2c). Further simulation details are provided in the SI (see section 4).

**Figure 3:**
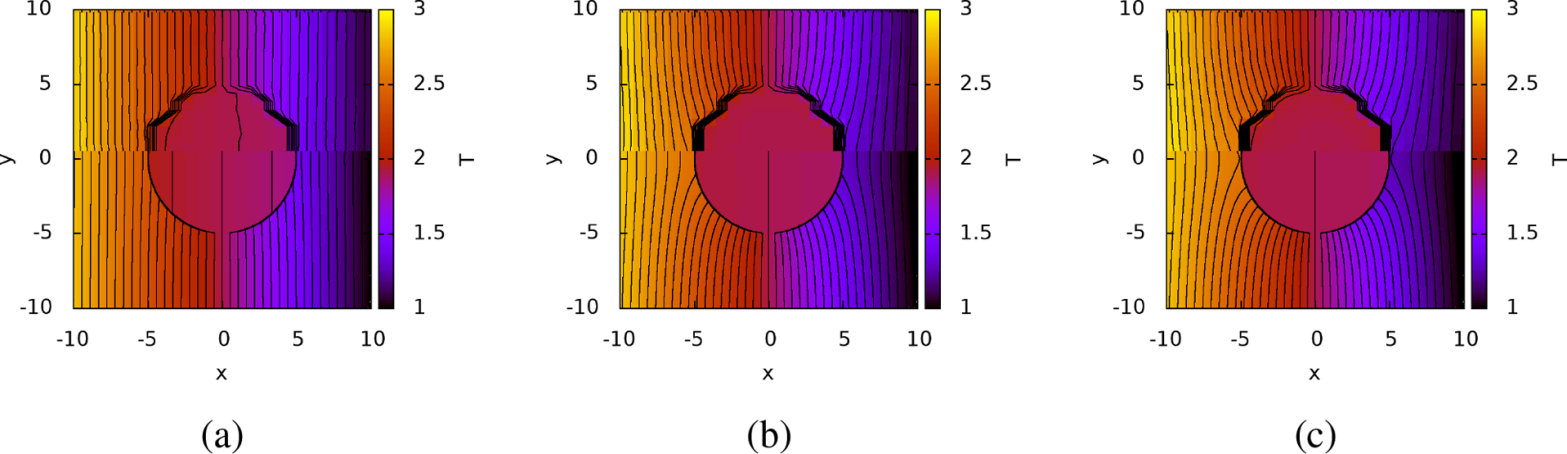
Overlay of the NEMD simulation (*y* > 0) and analytical results (*y* < 0), for ε_c_/ε_s_ = 20, 60, 100 corresponding to three ITCs, *G*_K,LJ_= 1.4 (a), 0.3 (b) and 0.11 (c) (in Lennard-Jones units, see SI), and colloid thermal conductivities of κ^c^ = 36 (a), κ^c^ = 28 (b) and κ^c^ = 20.5 (c) (see SI for details regarding calculations of the thermal conductivities). All the results were obtained using κ^s^ = 6.87, *T*_0_ = 1.9 and |∇*T*| = 0.09. To illustrate the different curvatures of the isotherms, we show in Figure S9 in the SI the temperature profile as a function of the angle, θ, at constant radial distance *r* = 5.5.

First, we compare the predicted temperature field around the spherical colloid against the simulated data using eqs 9 and 10 (see SI for details on the computation of the temperature field using NEMD simulations). We find good agreement between the predictions of the analytical solution and the NEMD results (see Figure 3), with some deviations at the colloid surface, connected to the granularity of the colloid surface. Away from the interfacial region, the agreement of the temperature field is good, supporting the accuracy of eqs 9 and 10. The impact of the ITC on the temperature field is also evident (c.f. Figure 3a–c). The field features stronger deformation with decreasing ITC, evident at distances >5 solvent molecular diameters. Analytical and simulation results indicate that ITC effects are needed to describe temperature fields around colloids.

We now discuss the theoretical predictions and simulated *S*_T_. The *S*_T_ computation using the setup in Figure 2a involves restraining the translational motion of the colloid with a spring, which results in a hydrodynamic flow^15,25^ around the colloid and a system size-dependent *S*_T_. For a given system size, the *S*_T_ is of the same order as the one obtained from the thermophoretic velocity of a freely drifting particle.^11^ We computed *S*_T_ for system sizes *L*_*y*,*z*_ = 16 and 20. To test the numerical results against eq 15, we used the colloid and solvent thermal conductivities corresponding to the bulk regions (see section 6 in the SI), the ITC obtained with the radial heat flux method (see Figure 2c), and the colloid radius, *R* = 5. We set a constant fitting parameter in eq 15, *c* = 4γ_T_χ_*L*_*y*,*z*__, where χ_*L*_*y*,*z*__ takes into account the finite size of the simulation box and its impact on *S*_T_.

**Figure 4:**
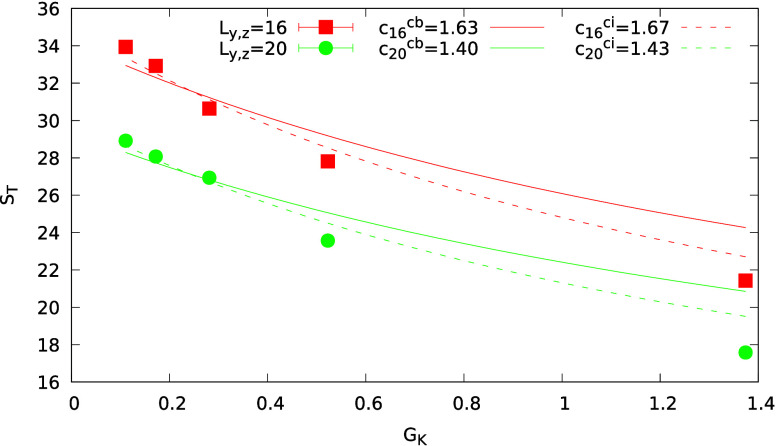
Soret coefficient as a function of *G*_K_ and system size. The solid lines represent fittings using the bulk fluid thermal conductivity (fitting parameter denoted by cb). The dashed lines represent fittings to eq 15 using the fluid interfacial thermal conductivity (fitting parameter denoted by ci). In both cases, the colloid core thermal conductivity was used.

Figure 4 shows the dependence of the simulated Soret coefficients with *G*_K_. The general dependence agrees with the theoretical results predicted by eq 15 and those shown in Figure 1c, namely, *S*_T_ decreases with increasing *G*_K_. We find that eq 15 reproduces the trends of the simulated *S*_T_, but some deviations are evident at large *G*_K_. We note that we used the bulk solvent thermal conductivities in the theoretical calculation. However, the density of the solvent next to the colloid differs significantly from the bulk density (see Figure S2). Hence, we recalculated the solvent thermal conductivity, including only the region within one molecular diameter from the colloid surface. The thermal conductivity of this layer is lower than the bulk one (see Figure S6 in the SI), and it leads to a better agreement between the simulation data and the theory (see Figure 4).

## Conclusions and Final Remarks

We have extended previous theories of the Soret effect,^6,36−38^ and presented the analytical solution to the temperature field around a spherical colloid, including the Kapitza resistance, to account for the temperature discontinuity at the colloid-solvent interface. In our formulation, the Soret coefficient varies with the deformation of the thermal field around the colloid, the thermal conductivities of colloid and solvent, and the ITC. The resulting equation includes corrected thermal conductivity terms to account for the ITC, *G*_K_. We show that the Soret coefficient depends strongly on ITC, for high colloid thermal conductivities (κ_c_ ≫ κ_s_), when *G*_K_ < 100–300 W/(K m^2^). These conditions can be found in gold nanoparticle suspensions. For κ^c^ ∼ κ^s^ (e.g., polymer nanoparticles), the impact of *G*_K_ is small, and for *G*_K_ → *∞*, we recover previous theories, which ignored *G*_K_ effects. We have verified the analytical equations using nonequilibrium molecular dynamics simulations of coarse-grained models of colloids immersed in a solvent. The simulated temperature fields agree with the “continuum” theory, and the Soret coefficient varies with *G*_K_ as predicted theoretically.

Eq 15 assumes a perfectly spherical surface and describes the thermophoretic forces, with constant thermal transport properties (thermal conductivity) right next to the particle surface. Our simulation model accurately reproduces the general theoretical predictions, despite the colloid size, which is not much larger than the solvent diameter, and the colloid atomistic nature, resulting in surface irregularities. Including interfacial effects by considering the thermal conductivity changes next to the colloid surface, brings the theoretical predictions into closer agreement with the simulation data. Considering that the simulated nanoparticle is not much larger than the diameter of the solvent, the agreement between theory and simulations is excellent. Additional work could involve investigating larger nanoparticles to systematically analyze the impact of surface granularity on the thermophoretic mobility.

Our work demonstrates the importance of nonequilibrium effects associated with explicit temperature gradients. Recent experiments^43^ suggested that thermophoresis is dominated by fluctuations at Peclet number (small thermal gradients), *Pe* = *RS*_T_∇*T* < 1, and by nonequilibrium transport for *Pe* > 1. The system investigated here is consistent with the *Pe* > 1 regime, where interfacial thermal gradients determine the thermophoretic motion. Furthermore, we find this nonequilibrium transport to dominate in a situation where the colloid is not much larger than the boundary layer.

We have demonstrated the importance of the ITC in the thermophoresis of colloidal particles. This contribution has been ignored before, but as shown here, it influences the temperature field around colloids and the thermophoretic force. Our theoretical analysis indicates that the ITC will modify the Soret coefficient in colloidal suspensions when the colloids feature high thermal conductivities, e.g. metallic nanoparticles. The functionalization of the colloids provides a route to tune the interfacial conductance maintaining a high thermal conductivity colloid/solvent ratio (see Figure 1). The impact of the ITC on the Soret coefficient should be considered in functionalized colloids with ITCs typical of hydrophilic materials, namely, 150–250 MW/(m^2^ K).

We anticipate that the ideas presented here will be helpful in advancing the description of thermophoresis, a very complex nonequilibrium coupling effect with potential applications to colloidal trapping, nanofluidics, and the design of analytical devices.

## Supporting Information Available

Additional equations and derivations discussed in the main text, simulation details, and additional simulation results (PDF)
