# Supplementary material for: Impact of the Interfacial Kapitza Resistance on Colloidal Thermophoresis
Source: ACS Omega. 2024 Oct 17;9(43):43779–84. doi: 10.1021/acsomega.4c06427 (PMC11525738; doi:10.1021/acsomega.4c06427)
Supplement: Supplementary file 1 — ao4c06427_si_001.pdf [file ao4c06427_si_001.pdf]

Impact of the Interfacial Kapitza Resistance on Colloidal Thermophoresis

Juan D. Olarte-Plata* and Fernando Bresme*

Department of Chemistry, Imperial College London, Molecular Sciences Research Hub Imperial College, W12 0BZ, London, United Kingdom

E-mail: j.olarte@imperial.ac.uk; f.bresme@imperial.ac.uk

1. Relevant Equations for the Temperature Profiles around Colloids in the Absence of Interfacial Thermal Conductance Effects

For spherical colloids in an external temperature field ∇T, of magnitude $|\nabla T|$ in the $\vec{x}$ direction, the temperature profile (in polar coordinates), far away from the particle is:

$$T^{ext} = T_0 + |\nabla T|\vec{x} = T_0 + |\nabla T| r \cos\theta \tag{S1}$$

where T_0 is a reference temperature far from the colloid. See the main text for definitions of the symbols below. Near the colloid, the solvent, $T^s(R,\theta)$ and colloid, $T^c(R,\theta)$ profiles fulfill the boundary conditions:

$$T^s(R,\theta) = T^c(R,\theta) \tag{S2}$$

$$\kappa^s \frac{\partial T^s(r,\theta)}{\partial r}\bigg|_{r=R} = \kappa^c \frac{\partial T^c(r,\theta)}{\partial r}\bigg|_{r=R} \tag{S3}$$

Following references,[1–3] the solution of the Laplace equation is:

$$T^s(r,\theta) = T_0 + |\nabla T| r \cos\theta \left[1 + \alpha \left(\frac{R}{r}\right)^3\right] \tag{S4}$$

$$T^c(r,\theta) = T_0 + |\nabla T| r \cos\theta \left[1 + \alpha\right] \tag{S5}$$

where R is the colloid radius. An explicit equation for α follows from Eq. (S3) above,

$$\alpha = \frac{\kappa^s - \kappa^c}{2\kappa^s + \kappa^c} \tag{S6}$$

2. Derivation of the Equations Discussed in the Main Text

We follow the approach of Würger to describe the relation between the surface stress and the Marangoni force for a freely drifting particle. The temperature gradient on the surface of the particle can be written as:[4]

$$\nabla_{\|}T = \left(\frac{1}{r}\frac{\partial T(r,\theta)}{\partial \theta}\right)\Bigg|_{r=R} = -|\nabla T|\sin\theta(1+\alpha)\mathbf{t} \tag{S7}$$

where $\mathbf{t}$ is the tangential vector at the surface. The Marangoni force is given by:[4]

$$\nabla_{\|}\gamma = \gamma_T \nabla_{\|}T \tag{S8}$$

where the Marangoni parameter is defined as the derivative of the interfacial free energy, γ, with respect to the temperature, $\gamma_T = d\gamma/dT$. The dissipative part of the stress tensor, σ', is given by:[4]

$$\sigma'_{rr} = 2\eta\frac{\partial \hat{v}_r}{\partial r} \tag{S9}$$

$$\sigma'_{r\theta} = \eta\left(\frac{1}{r}\frac{\partial \hat{v}_r}{\partial \theta} + \frac{\partial \hat{v}_\theta}{\partial r} - \frac{\hat{v}_\theta}{r}\right) \tag{S10}$$

where η is the viscosity of the fluid. $\hat{v}_r$ and $\hat{v}_\theta$ are the radial and tangential components of the fluid velocity field near the particle, in the particle frame of reference.

For a particle drifting with velocity $\mathbf{u} = u\hat{x}$ (due to the thermophoretic force), the solution to the Stokes equation for an incompressible fluid, $\eta\nabla^2\hat{\mathbf{v}} = \nabla P$, can be written as:[4]

$$\hat{v}_r = -u\cos\theta\left(1-\frac{R^3}{r^3}\right) \tag{S11}$$

$$\hat{v}_\theta = u\sin\theta\left(1+\frac{R^3}{2r^3}\right) \tag{S12}$$

For this case, the tangential component of the stress tensor evaluated at the particle surface is:

$$\sigma'_{r\theta}|_{r=R} = \frac{-3\eta u\sin\theta}{R} \tag{S13}$$

The thermophoretic velocity of the particle can be found by balancing the tangential component of the stress tensor with the Marangoni force, $\sigma'_\theta \mathbf{t} = -\gamma_T \nabla_\parallel T$,[4] giving:

$$\frac{-3\eta u\sin\theta}{R} = \gamma_T|\nabla T|\sin\theta(1+\alpha) \tag{S14}$$

The drift velocity of the particle can thus be written as:

$$u = -\frac{\gamma_T R(1+\alpha')}{3\eta}|\nabla T| \tag{S15}$$

3. Forces on a Fixed Particle

In our simulations, the translational motion of the nanoparticle is restrained in the middle plane between the hot and cold thermostats. This physical situation corresponds to that of a fixed particle and results in a velocity field distinctively different from the one obtained with the freely drifting particle. However, the corresponding friction force is the same except for a constant. Below, we derive the relevant equations for this case.

The total force on the particle is given by[5]

$$F_{tot} = \oint_S (-p\cos\theta + \sigma'_{rr}\cos\theta - \sigma'_{r\theta}\sin\theta)dA \tag{S16}$$

where p is the pressure, and σ'_{ij} are components of the stress tensor.

The general solution for the fluid velocity around a spherical particle is given by:[5]

$$v_r = u_0\cos\theta\left(1 - \frac{2a}{r} + \frac{2b}{r^3}\right) \tag{S17}$$

$$v_\theta = -u_0\sin\theta\left(1 - \frac{a}{r} - \frac{b}{r^3}\right) \tag{S18}$$

where the constants a and b are determined from the boundary conditions. For instance, a freely drifting particle with *stick* boundary conditions implies that at $r = R$, $v_r = 0$ and $v_\theta = 0$. The solution is given by $a = 3R/4$ and $b = R^3/4$, which implies:

$$\hat{v}_r = u_0\cos\theta\left(1 - \frac{3R}{2r} + \frac{R^3}{2r^3}\right) \tag{S19}$$

$$\hat{v}_\theta = -u_0\sin\theta\left(1 - \frac{3R}{4r} - \frac{R^3}{4r^3}\right) \tag{S20}$$

For the fixed particle, the fluid velocity can be written as:[6]

$$v_r = u_0\cos\theta\left(\frac{R}{r} - \frac{R^3}{r^3}\right) \tag{S21}$$

$$v_\theta = -\frac{u_0\sin\theta}{2}\left(\frac{R}{r} + \frac{R^3}{r^3}\right) \tag{S22}$$

In this case, u_0 represents the velocity of the fluid at the particle's surface. With these expressions for the velocity field, we arrive to:

$$\sigma'_{rr}|_{r=R} = 4\eta \frac{u_0}{R} \cos\theta \tag{S23}$$

$$\sigma'_{r\theta}|_{r=R} = 3\eta \frac{u_0}{R} \sin\theta \tag{S24}$$

We note that the tangential stress, $\sigma'_{r\theta}$, has the same solution as the freely drifting particle with surface forces (see section 2 in the SI). This justifies using the approach proposed in Ref.[4] when equating the tangential stress to the Marangoni force.

The corresponding contributions to the total force are given by:

$$F_{\sigma'_{rr}} = \frac{16\pi}{3} \eta R u_0 \tag{S25}$$

$$F_{\sigma'_{r\theta}} = -8\pi\eta R u_0 \tag{S26}$$

The pressure contribution to the total force can be found from the following expression:[4]

$$P = P_0 + 2\alpha \cos\theta \frac{\eta u_0 R}{r^2} \tag{S27}$$

where $\alpha = 1/2$ corresponds to the solution for the fluid velocity field around the fixed particle. The pressure contribution to the total force thus reads:

$$F_P = -\frac{4\pi}{3} \eta R u_0 \tag{S28}$$

The total force on the particle is thus given by:

$$F_{total} = F_{\sigma'_{rr}} + F_{\sigma'_{r\theta}} + F_P = -4\pi\eta R u_0 \tag{S29}$$

From the previous equation, we note that the friction coefficient of the fixed particle with surface forces has a factor of 4π, as discussed in the main text.

4. Simulation Details

The fluid, with reduced density $\rho = \rho_n \sigma^3 = 0.8$ (ρ_n is the number density in particles/m^{-3}) was described using the WCA model, *i.e.* a Lennard-Jones 12-6 potential, $4\varepsilon_s \left[(\sigma_s/r)^{12} - (\sigma_s/r)^6\right]$, with a cut-off radius corresponding to the minimum of the potential, *i.e.* $r_c = r_0 = 2^{1/6}\sigma$, with $\epsilon_s = 1.0$. σ is the diameter of the solvent and the particles inside the colloid. We used reduced units, $r = r_{SI}/\sigma$, $T = k_B T_K/\varepsilon_s$, where r_{SI} is the distance in SI units, ε_s is the interaction strength between solvent particles and T_K the temperature in Kelvin. in these units the ITC is defined as, $G_{K,LJ} = (G_K \sigma^2 \sqrt{m\sigma^2/\varepsilon_s})/k_B$. All the solvent-particle interactions were computed using the WCA model. The interactions between the particles inside the colloids were described using a strongly attractive Lennard-Jones potential with interaction strength ε_s and a cut-off radius $r_c = 2.5\sigma$.

The simulations were run with a timestep $\delta t = 0.0025$ in Lennard Jones units, and using LAMMPS.[7] After 10^4 timesteps in the NVT ensemble with $T = 1.0$, the thermostats (see Figure S1) were activated. The first 10^5 timesteps were discarded before sampling for an additional 10^7 timesteps. The results reported in our work were obtained using 20 independent replicas, starting from random atomic velocities and colloid orientations. To perform the simulations using the two setups (see Fig. S1), we generated cubic (*radial heat flux*) or rectangular (*external gradient*) simulation boxes with volume $V = (16a_0) \times (16a_0) \times (16a_0)$ or $V = (32a_0) \times (16a_0) \times (16a_0)$, where $a_0 = 2^{2/3}\sigma$ is the FCC lattice parameter of a solid with $\rho^* = 1.0$. To investigate finite-size effects in the computations of the Soret coefficient, we also simulated systems with $L_{y,z} = 20a_0$ for the *external gradient* set-up. In all cases, $L_x = 2L_{y,z}$. To set a temperature gradient, hot and cold thermostatting regions were defined at the center and edges of the simulation box. The thermostatting regions correspond to a spherical core, $r < 2.0\sigma$, and shell, $R > 9.7\sigma$ (*radial heat flux*), or rectangular slabs of width $4a_0$ (*external gradient*). Within these regions, the velocities of the fluid particles were rescaled to the target temperatures every 100 timesteps. The center-of-mass momentum was also removed with the same frequency to prevent a drift in the translational motion of the

atoms. The *external gradient* configuration generates two temperature gradients in opposite directions (see Figure 2 in the main paper or Figure S1). A spherical particle, cut from an FCC lattice with density $\rho = 1.0$ and radius $R = 5\sigma$, was placed in the center of each compartment and attached with harmonic potential, given by $U(r) = 0.5\ k(r - r_0)^2$, with force constant $k = 10^3$. Snapshots of the simulation box and typical temperature profiles are shown in Fig. S1. All the visualizations were done using OVITO.[8]

The temperature fields were calculated by dividing the simulation box in cubic voxels of volume $\sim (0.79\sigma)^3$, where the local temperature was sampled. To exploit the symmetry of the problem and improve sampling, the data was re-binned (using a weighted average) to (x, r) coordinates, where $r = \sqrt{y^2 + z^2}$. The bin size for the r coordinate was $\sim 0.56\sigma$. The contours were generated using a linear interpolation, with increasing levels of 0.05.

5. Density Profiles of the Fluid

Fig. S2 shows the radial density profile of the fluid as a function of radial distance to the center of geometry of the colloid and the colloid interaction strength, obtained under equilibrium conditions at $T = 2.0$. The results in Fig. S2 show that the colloid interactions do not impact the density profiles of the solvent surrounding the colloid.

6. Calculation of the Colloid-Solvent Interfacial Thermal Conductance, G_K

To compute the interfacial thermal conductance G_K we performed simulations targeting the highest and lowest interfacial temperatures obtained in the simulations of the Soret coefficient (see Fig. S1). Our simulations also consider the rectification of the G_K, namely, the solvent is hotter or colder on both sides of the colloid (see Fig. S1).

We now discuss the dependence of the Soret coefficient on the ITC. To address this point,

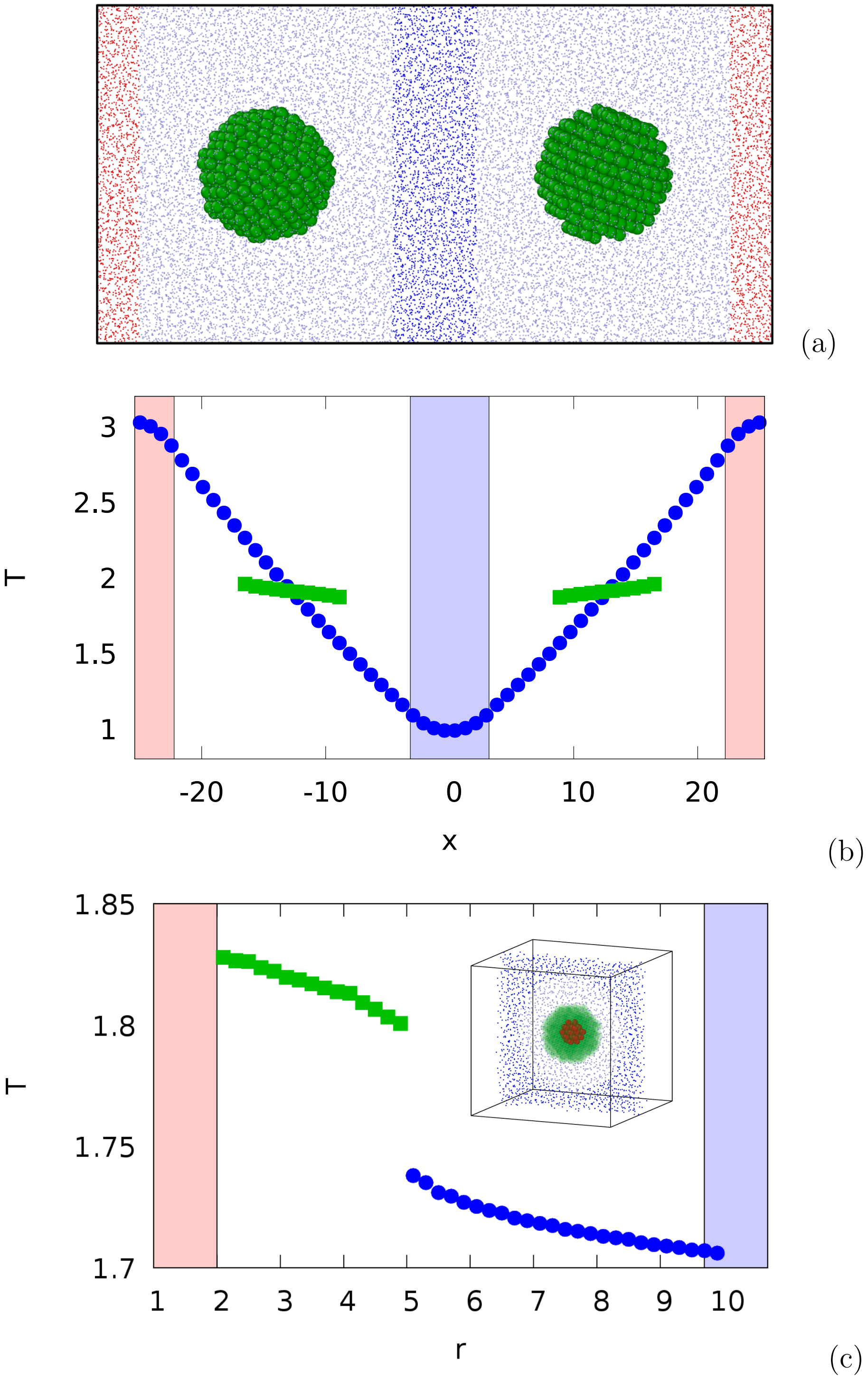

Figure S1: (a) Snapshot of the simulation box in the *external gradient* set-up, showing the thermostatting regions (red-hot, blue-cold). The solvent particles are represented as blue dots for visualization purposes, and the colloid as green spheres. (b) Temperature profile for a system with $\epsilon_c/\epsilon_s = 20$, showing the temperature of the fluid (blue circles) and the colloid (green squares). (c) Temperature profile for the *radial heat flux* system with $\epsilon_c/\epsilon_s = 20$, showing the temperature of the fluid (blue circles) and the spherical particle (green squares), and the corresponding temperature jump. The inset shows a snapshot of the simulation box, highlighting the thermostatted core (red) and the surrounding solvent (blue dots).

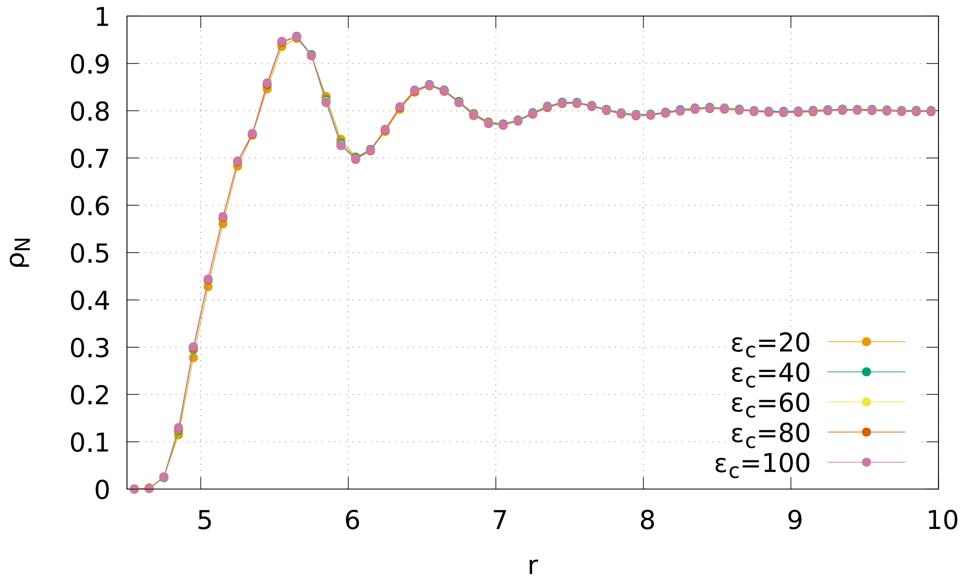

Figure S2: Solvent radial density profiles around the colloid, as a function of the colloid interaction strength. The profiles were obtained at equilibrium conditions and $T = 2.0$.

we vary G_K, keeping the solvent-particle interaction strength constant and approximately the same interfacial temperature; hence, γ_T does not change with G_K. This notion is supported by the lack of dependence of the colloid solvation structure with ε_c (see Fig. S2). By modifying the colloid particle-particle interactions, we isolate G_K and surface tension effects. This leads to a systematic change of the colloid vibrational density of states (VDoS) relative to the VDoS of the solvent. By changing the overlap of the two VDoS we achieve higher or lower G_K, and we can systematically test Eq. (9) in the main paper. Figure S3 shows the impact of the ε_c on the overlapping of the colloid and solvent Vibrational Density of States (VDoS). The ITC decreases with increasing ε_c due to a shift of the colloid VDoS to higher frequencies as ε_c increases (see Fig. S4).

The VDoS was computed as the Fourier transform of the velocity autocorrelation function for the fluid and solid particles:

$$VDoS_{j,k}(\omega) = \int_0^\infty \frac{\langle v_{j,k}(0) v_{j,k}(t) \rangle}{\langle v_{j,k}(0) v_{j,k}(0) \rangle} \exp(-2\pi i \omega t)\, dt \tag{S30}$$

where $\langle v_{j,k}(0) \cdot v_{j,k}(t) \rangle$ is the velocity autocorrelation function of particle type j and com-

ponent k. We used a simulation box equivalent to Fig. S1a with no temperature gradient and average temperature $T = 2.0$. The velocity autocorrelation function for fluid and colloid particles was calculated for 100 windows of 2500 timesteps each (using $\delta t = 0.001$) and averaged over ten replicas. All the colloid particles were included in the calculation.

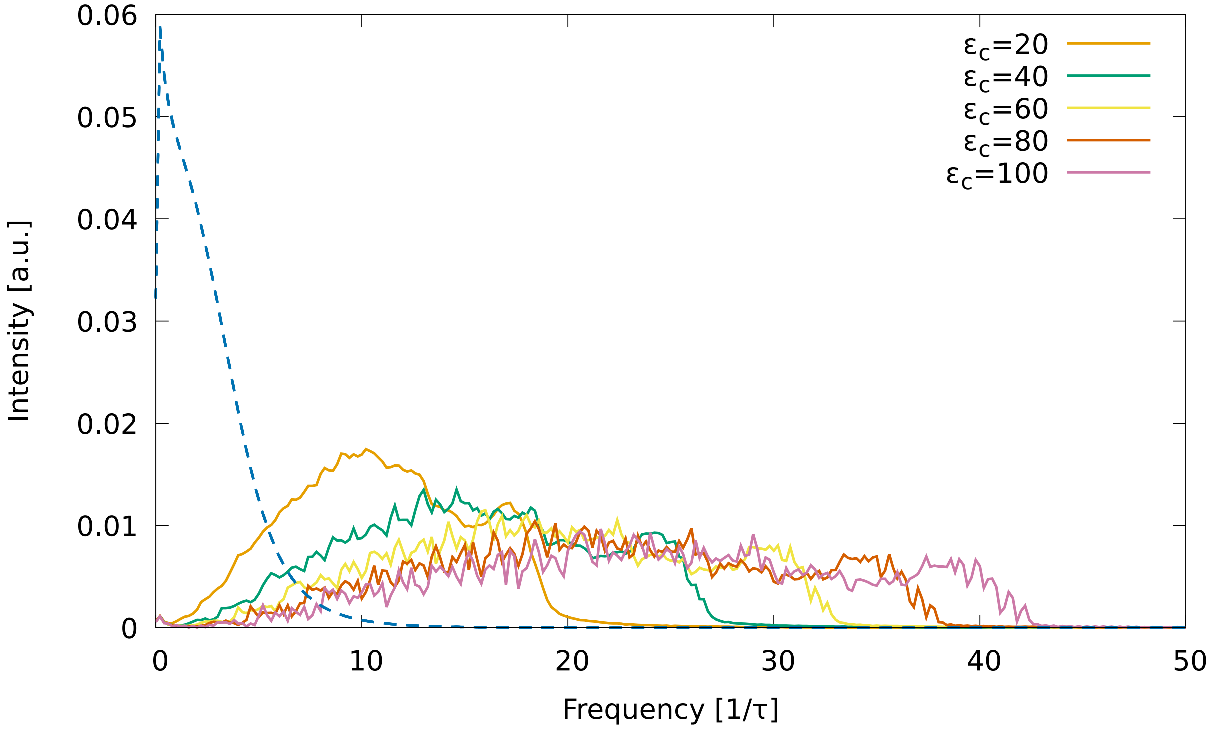

Figure S3: (a) VDoS of the colloid and solvent as a function of ε_c. The fluid VDoS is represented with a blue dashed line.

We compute the interfacial thermal conductance of uniform nanoparticles as a function of their internal interaction strength using the *radial heat flux* simulation set-up (see Fig. S1). The temperature of the colloid core is thermostatted to a target temperature $T_c = 1.9$. The temperature of the bulk solvent is varied between $T_s = 1.7$ and $T_s = 2.1$ to account for the colloid-solvent contact on the hot and cold regions (see Fig. S1). For the calculation of the temperature "jump", we fitted the temperature profiles using the heat diffusion equation near the interfacial region (see Fig. S5). The range of radial distance for the colloid was restricted to $r_c^* = [4.0, 5.0]$, while for the solvent we used the interval $r_s = [5.0, 6.0]$.

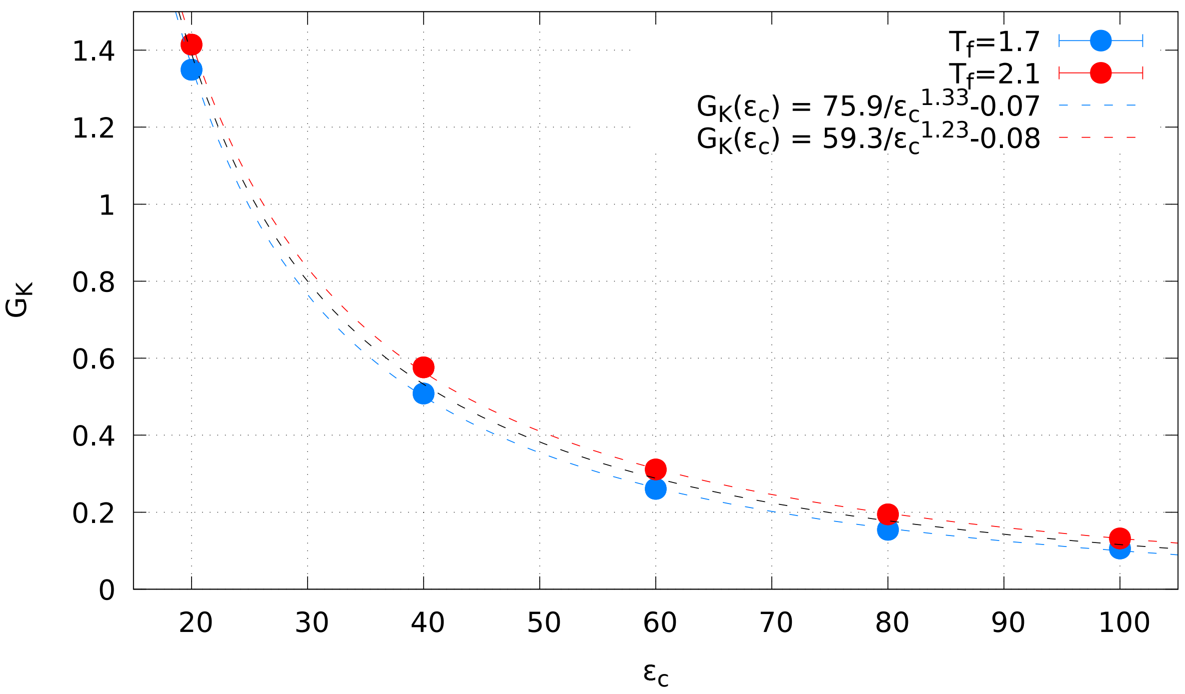

Figure S4: Interfacial thermal conductance as a function of the colloid internal interaction strength for different bulk fluid temperatures. The dashed lines indicate fittings to a function of the form $G(\epsilon_c) = a/\epsilon_c^b + d$.

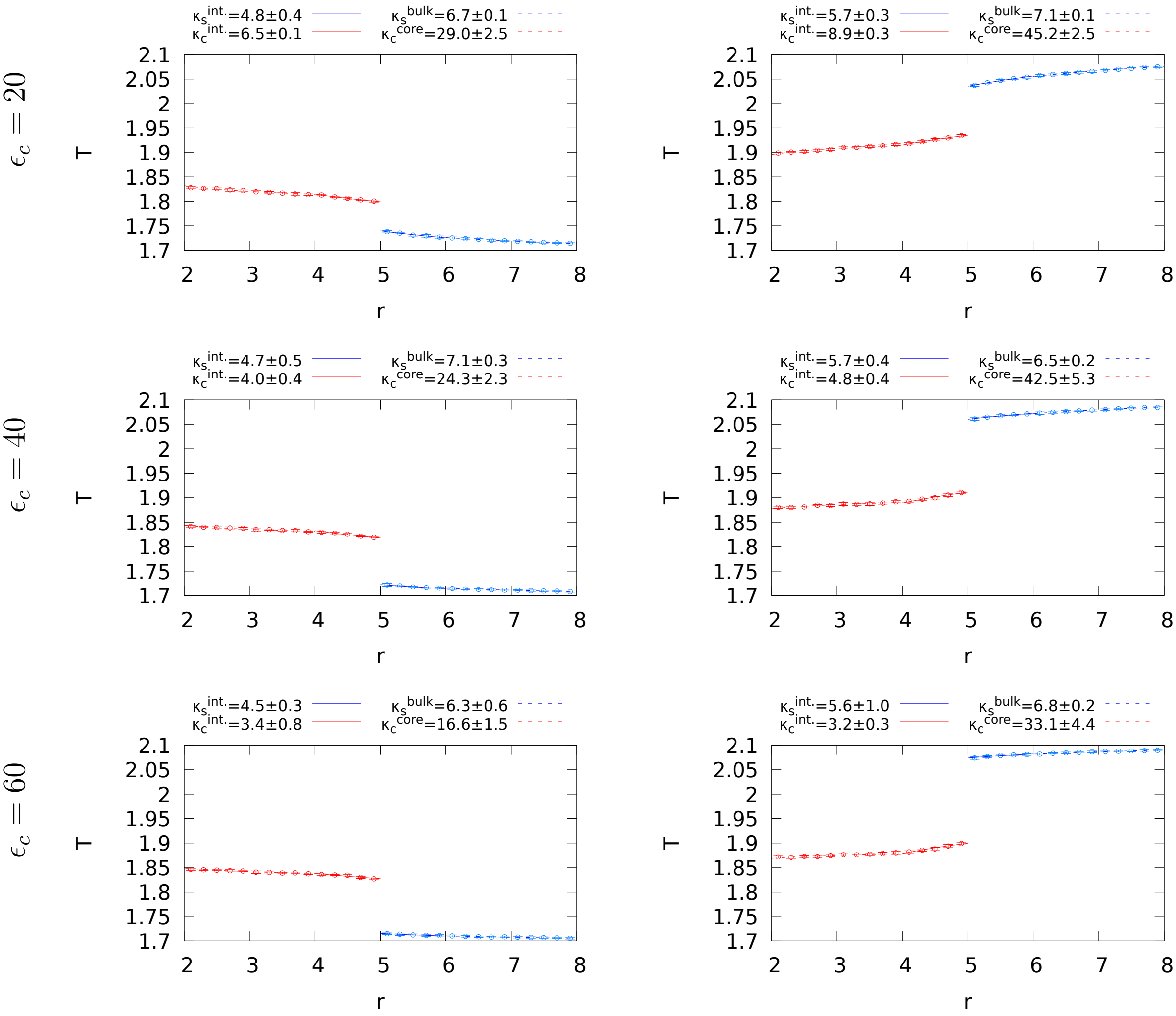

Figure S5: Temperature profiles in the *radial heat flux* configuration, for different boundary conditions and colloid interaction strengths. The lines represent the fitting used to calculate the thermal gradient and thermal conductivities. κ_α^β refers to the colloid ($\alpha = c$) or solvent ($\alpha = s$) thermal conductivity obtained using the $\beta =$"bulk" for the solvent, $\beta =$"core" for the colloid or interfacial region $\beta =$"int" for the solvent. Error bars represent the standard deviation calculated from the individual temperature profiles of 10 replicas.

6. Thermal Conductivity of the Solvent and Colloid

The thermal conductivity of the solvent was calculated using the *radial heat flux* simulation set-up (see Fig. S1). Due to the decreasing heat flux with increasing particle interaction strength, we focused on the range $\varepsilon_c = 20 - 60$. Fig. S6 shows the thermal conductivity as a function of the fitting region and the boundary condition of the fluid. The thermal conductivity does not feature a significant variation with either temperature or particle interaction strength ε_c, Hence, to plot the theoretical curves in Fig. 3 in the main text, we used the thermal conductivities $\kappa_s^{bulk} = 6.87$ and $\kappa_s^{int.} = 5.16$ (see dashed horizontal lines in Fig. S6).

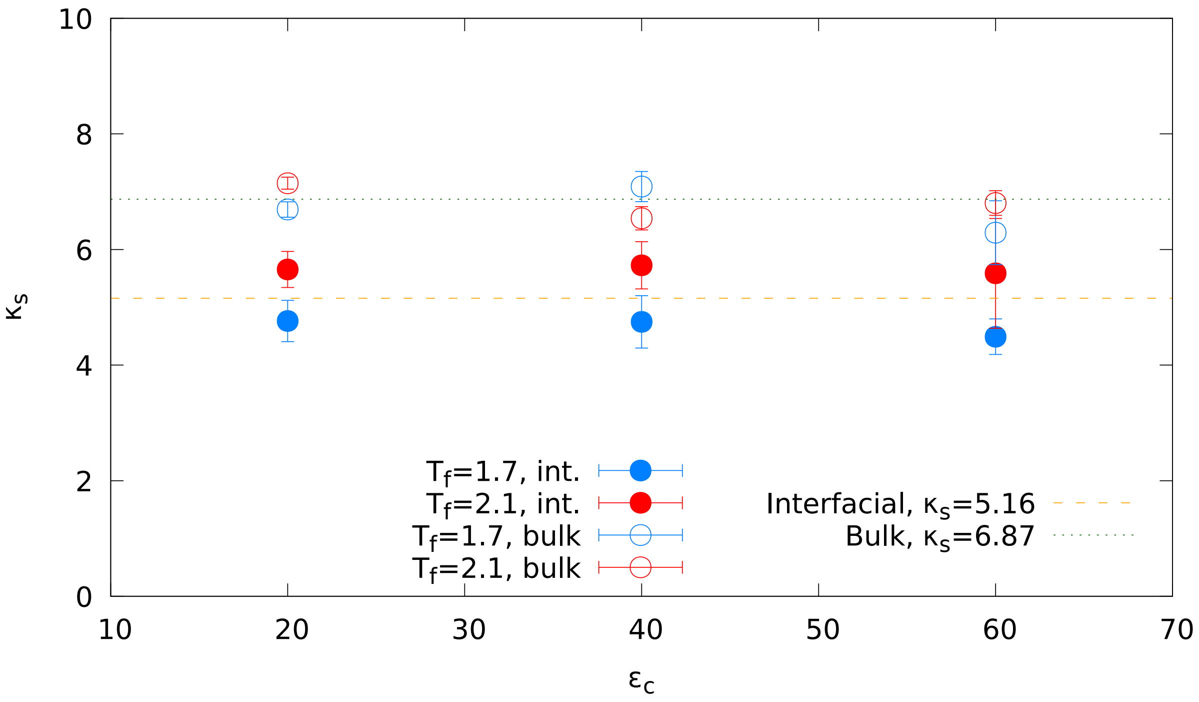

Figure S6: Thermal conductivity of the solvent as a function of the colloid interaction strength and the temperature. The upper values were obtained using the temperature gradient in the bulk region and the lower values were obtained using the interfacial temperature gradient (see Fig. S5 for definitions of bulk and interfacial regions). Red and blue points represent the thermal conductivity at different temperatures, targeting the temperatures at the hot or cold side of the colloid surface (see Figure S1). The dashed horizontal lines represent the average thermal conductivities for each temperature and varying ϵ_c.

The thermal conductivity of the colloid (see Fig. S7) was calculated using the *radial heat flux* simulation set-up. We used Fourier's law by fitting the temperature gradient inside the colloid in an interval far from the colloid surface to avoid including interfacial effects (the interval $r_s = [2.0, 4.0]$).

Error bars for the thermal conductivities represent the asymptotic standard error from the fitting of the temperature profiles (and their associated standard deviations) shown in Fig. S5.

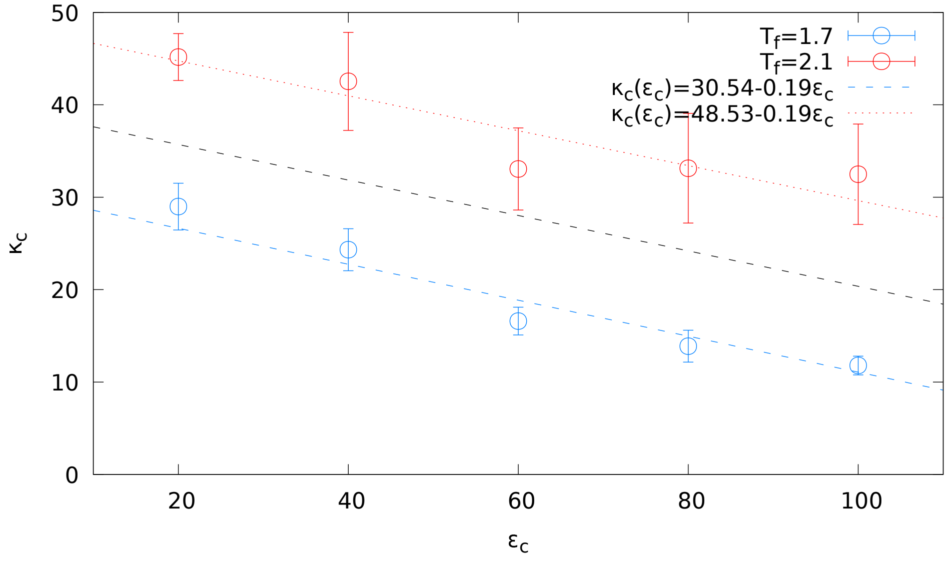

Figure S7: Thermal conductivity of the colloid as a function of the colloid interaction strength for different temperatures. The dashed lines indicate fittings to a linear function. The black dashed lines represent the average of both high and low-temperature lines.

7. Estimation of the Effective Thermal Conductivities of Functionalized Colloids

We used the following approach to estimate the effective thermal conductivity of the functionalized colloids discussed in Fig. 1c in the main paper. The effective thermal conductivity of the colloid, κ_{eff}, can be estimated using the model introduced by Hasselman and Johnson,[9] which reduces to the expression derived by Maxwell for infinite interfacial thermal

conductance. As an example we consider here a gold colloid with $\kappa_{Au} \sim 300$ W/(K m), coated with an alkanethiol monolayer with $\kappa_m \sim 0.45$ W/(K m)[10] at ~ 300 K. The interfacial thermal conductance Au-monolayer interface is ~ 800 MW/ (K m^2).[10] Following ref.[9] the effective thermal conductivity of the colloid is given by:

$$\kappa_{eff} = \kappa_m \frac{2\left(\frac{\kappa_{Au}}{\kappa_m} - \frac{\kappa_{Au}}{RG_K} - 1\right) V_{Au} + \frac{\kappa_{Au}}{\kappa_m} + \frac{2\kappa_{Au}}{RG_K} + 2}{\left(1 - \frac{\kappa_{Au}}{\kappa_m} + \frac{\kappa_{Au}}{RG_K}\right) V_{Au} + \frac{\kappa_{Au}}{\kappa_m} + \frac{2\kappa_{Au}}{RG_K} + 2} \tag{S31}$$

where $V_{Au} = (R/(R+d))^3$ is the volume fraction of the gold particle with radius R, relative to the total volume of the colloid, defined by the radius $R + d$, where d is the thickness of the monolayer coating the gold core.

Considering a colloid of radius R =250 nm and monolayers of thickness between 1 or 2 nm, we obtain the effective thermal conductivities 58.6 W/(K m) or 38.8 W/(K m), respectively. Ignoring the interfacial thermal conductance, $G_K \to \infty$, we find that the effective thermal conductivity is higher, 83.0 and 48.2 W/(K m) for monolayer thicknesses of 1 or 2 nm, respectively.

8. Soret Coefficient vs. Internal Interaction Strength and System Size

Fig. S8 shows the Soret coefficients obtained with the method discussed in section 2 above (see also discussion in the main paper pages 3 and 4). We considered different colloid interaction strengths as well as different system sizes. The Soret coefficient decreases with increasing system size (see Fig. S8 and also the discussion on the truncation of the hydrodynamic field in reference[11]).

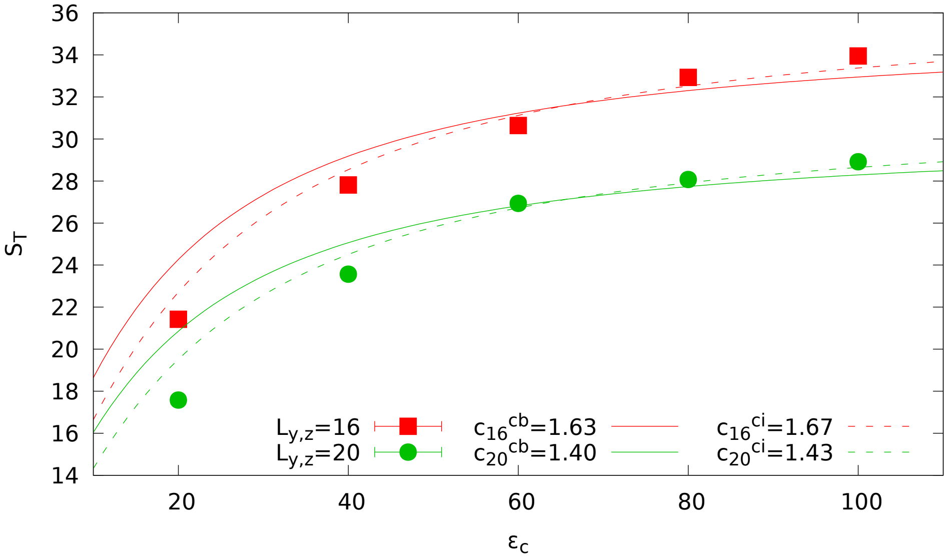

Figure S8: Soret coefficient as a function of the particle interaction, ϵ_c, and system size. The symbols represent simulation results, and the lines fittings to the theoretical equations. See the discussion in the main text.

9. Angular Temperature Profiles

Figure S9 shows the angular temperature profile at constant radius 5.5 and different ε_c values.

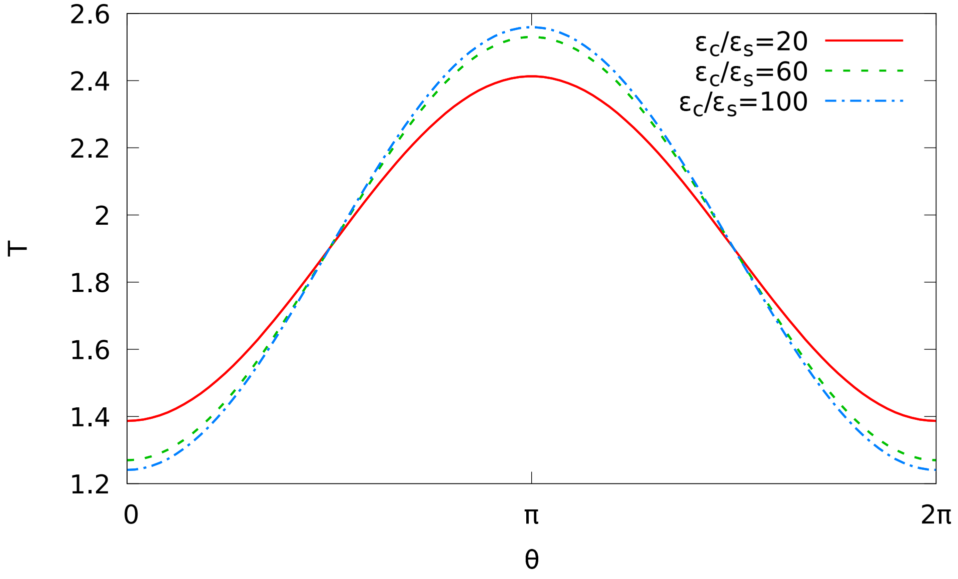

Figure S9: Angular temperature profiles for the fluid near the particle (at radius 5.5).

References

(1) Giddings, J.; Shinudu, P. M.; Semenov, S. N. Thermophoresis of Metal Particles in a Liquid. *Journal of Colloid and Interface Science* **1995**, *176*, 454–458.

(2) Bickel, T.; Zecua, G.; Würger, A. Polarization of active Janus particles. *Phys. Rev. E* **2014**, *89*, 050303.

(3) Gaspard, P.; Kapral, R. The stochastic motion of self-thermophoretic Janus particles. *Journal of Statistical Mechanics: Theory and Experiment* **2019**, *2019*, 074001.

(4) Würger, A. Thermophoresis in Colloidal Suspensions Driven by Marangoni Forces. *Phys. Rev. Lett.* **2007**, *98*, 138301.

(5) Landau, L. D.; Lifshitz, E. M. *Fluid Mechanics, Second Edition: Volume 6 (Course of Theoretical Physics)*, 2nd ed.; Course of theoretical physics / by L. D. Landau and E. M. Lifshitz, Vol. 6; Butterworth-Heinemann, 1987.

(6) Morthomas, J.; Würger, A. Hydrodynamic attraction of immobile particles due to interfacial forces. *Phys. Rev. E* **2010**, *81*, 051405.

(7) Plimpton, S. Fast Parallel Algorithms for Short-Range Molecular Dynamics. *Journal of Computational Physics* **1995**, *117*, 1–19.

(8) Stukowski, A. Visualization and analysis of atomistic simulation data with OVITO-the Open Visualization Tool. *MODELLING AND SIMULATION IN MATERIALS SCIENCE AND ENGINEERING* **2010**, *18*.

(9) Hasselman, D.; Johnson, L. F. Effective Thermal Conductivity of Composites with Interfacial Thermal Barrier Resistance. *Journal of Composite Materials* **1987**, *21*, 508–515.

(10) Olarte-Plata, J. D.; Gabriel, J.; Albella, P.; Bresme, F. Spatial Control of Heat Flow at the Nanoscale Using Janus Particles. *ACS Nano* **2022**, *16*, 694–709.

(11) Bresme, F.; Olarte-Plata, J. D.; Chapman, A.; Albella, P.; Green, C. Thermophoresis and thermal orientation of Janus nanoparticles in thermal fields. *Eur. Phys. J. E* **2022**, *45*, 59.
